# Bacillus of Tubercle

**Published:** 1884-02-20

**Authors:** 


### *BACILLUS OF TUBERCLE.*

We notice that Dr. Austin Flint, in a paper in the Medical News, expresses his belief in the germ theory of tubercular consumption. He arrived at this conclusion after numerous experimental observations, aided by a microscopical expert.

